# Correlates of Healthy Aging in Geriatric HIV (CHANGE HIV)—CTN 314

**DOI:** 10.3390/v15020517

**Published:** 2023-02-13

**Authors:** Alice Zhabokritsky, Rosemarie Clarke, Ron Rosenes, Graham Smith, Mona Loutfy, Nisha Andany, Julian Falutz, Marina Klein, Marianne Harris, Silvia Guillemi, Darrell H. S. Tan, Gordon Arbess, Sharon Walmsley

**Affiliations:** 1 Toronto General Research Institute, University Health Network, University of Toronto, Toronto, ON M5G 2M9, Canada; 2 Maple Leaf Medical Clinic, 14 College St, Toronto, ON M5G 1K2, Canada; 3 Women’s College Research Institute, Women’s College Hospital, Toronto, ON M5S 1B2, Canada; 4 Sunnybrook Health Science Centre, University of Toronto, Toronto, ON M4N 3M5, Canada; 5 Chronic Viral Illness Service, McGill University Health Centre, Montreal, QC H4A3J1, Canada; 6 Infectious Diseases and Immunity in Global Health Program, Research Institute of McGill University Health Centre, Montreal, QC H4A 3J1, Canada; 7 BC Centre for Excellence in HIV/AIDS, University of British Columbia, 608-1081 Burrard Street, Vancouver, BC V6Z 1Y6, Canada; 8 Department of Family Medicine, University of British Columbia, Vancouver, BC V6T 1Z3, Canada; 9 St. Michael’s Hospital, University of Toronto, Toronto, ON M5B 1W8, Canada

**Keywords:** HIV/AIDS, aging, cohort, health

## Abstract

The Correlates of Healthy Aging in Geriatric HIV (CHANGE HIV) study, CTN 314, is the first Canadian cohort of people living with HIV aged 65 years and older. The cohort was established with the purpose of characterizing the multidimensional health status of this population and identifying factors influencing healthy aging. The study builds on the World Health Organization (WHO) Aging and Health conceptual framework, generating a comprehensive profile of health domains (physical, social, mental health, cognitive function, and quality of life), health determinants (biologic, personal, and environmental), and HIV-specific factors that may interact with and influence health in people aging with HIV. The data for the first 353 participants are presented, focusing on sociodemographic factors, comorbidities, coinfections, frailty, cognitive function, loneliness, and resilience using a sex/gender stratified analysis. The cohort thus far is 91% men and the median age is 70 years (range from 65 to 85). Several vulnerabilities were observed, including a high prevalence of comorbidities and frailty. Women especially faced financial insecurity and precarious social structures; a large proportion live alone and only 6% are married or in steady relationships. Identifying strategies to address these vulnerabilities will empower people aging with HIV to optimize their health, quality of life, and independence.

## 1. Introduction

At the beginning of the HIV epidemic, the average age of people diagnosed with HIV was in the mid 20’s and the mean survival was less than 1 year [1]. At that time, people living with HIV had no expectation of living into older age and their ill health led many to resign from their jobs, deplete their financial resources, and abandon their life goals and hope of a meaningful future. Despite these difficult beginnings, those who managed to survive and access effective combination antiretroviral therapy (ART) are now entering into their senior years and now represent a large proportion of the estimated 62,790 people who live with HIV in Canada [2]. In addition, 1 in 5 new HIV infections are occurring among people over the age 50. It is expected that, in the next decade, the median age of persons living with HIV in resource-rich countries including Canada will reach greater than 65 years [3].

**Figure 1:**
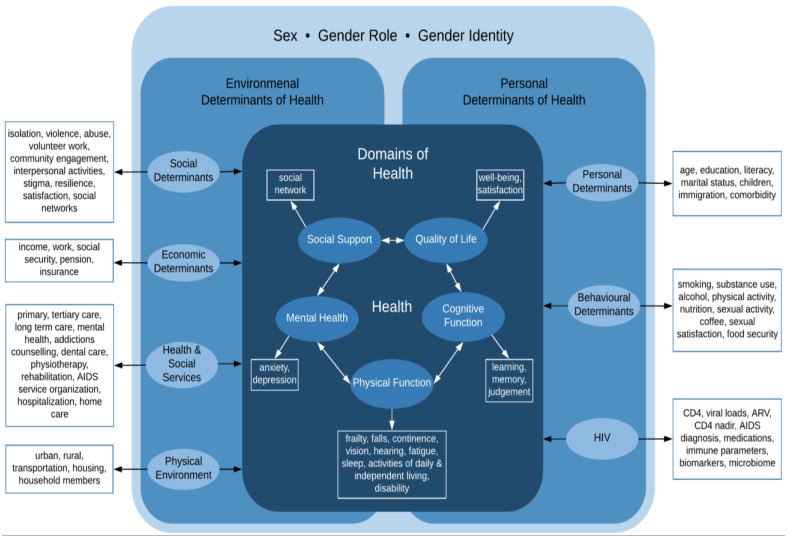
Conceptual model of healthy aging used in the CHANGE HIV study design.

As the life expectancy among persons living with HIV approaches that of the general population, persons living with HIV and their caregivers are facing a whole new set of challenges [4]. What is the experience of aging with HIV? What strategies will support persons living with HIV to age well? In the general population, women tend to live longer but with more comorbidity; will this be the case for women living with HIV? Does the health experience of aging with HIV differ depending on sex and gender identity? Critical knowledge gaps exist on how HIV and its therapies impact and interact with normal aging. Understanding these dynamics is essential for health care professionals, clinics, hospitals, long-term care (LTC) facilities, and AIDS service organizations to anticipate the challenges of aging with HIV. In Canada, the health care system is publicly funded, where universal access to care includes prescription drug coverage for individuals aged 65 years and older, including antiretroviral therapy. Most people living with HIV are receiving care from primary care physicians with expertise in HIV care or specialty HIV clinics. However, there are no geriatric models of care for people living with HIV in Canada across hospital, clinic, and LTC settings to address the complex social and health care needs of this population. The Correlates of Healthy Aging in Geriatric HIV (CHANGE HIV) study is the first Canadian cohort of PLWH aged 65 years and older. The cohort was established in 2019 with the purpose of advancing knowledge in HIV and aging by characterizing the multidimensional health status of persons living with HIV in Canada aged 65 years and older and identifying factors influencing healthy aging. The study design builds on the WHO conceptual framework for Healthy Aging, which aims to comprehensively capture the health status of persons living with HIV (Figure 1) [5]. Health status, the dependent variable in the CHANGE HIV study, is defined by measures from health domains including physical function, cognitive function, mental health, quality of life, and social support (shown in diamonds). These are conceptualized in the context of personal (behavioral and physical) and environmental (economic, social, physical environment, and health services) determinants of health (shown in circles), including HIV-specific factors (immune, viral, therapy, and complications). Each domain and determinant of health is operationalized by several independent variables measured in the cohort (shown in rectangles). We present the cohort profile for the first 353 participants enrolled in the study with a focus on sociodemographic factors, comorbidities, coinfections, frailty, cognitive function, loneliness, and resilience using a sex/gender stratified analysis.

## 2. Methods

### 2.1. Study Administration

CHANGE HIV is a collaborative, interdisciplinary study. The study team is composed of clinicians, researchers, trainees, persons living with HIV, and representatives from community organizations dedicated to advancing healthy aging among persons living with HIV. In keeping with the principals of GIPA (greater involvement of persons living with HIV), MIWA (meaningful involvement of women living with HIV), and MEPA (meaningful engagement of persons living with HIV), the community advisory board of those living and aging with HIV has been involved in the study from the outset. The CHANGE HIV sex and gender champion team of academics and community members ensures these crucial variables are considered in every aspect of the study.

### 2.2. Source Population, Eligibility, and Recruitment

The source population for the cohort is persons diagnosed with HIV, aged 65 years and older, who live in Canada and actively receive HIV care at one of seven participating study sites. The sites comprise primary and tertiary clinics across three Canadian provinces (British Columbia, Ontario, and Quebec). These provinces have the highest prevalence of persons living with HIV and together account for over 80% of the total population of persons living with HIV in Canada [2]. The study sites provide care to nearly 11,000 persons living with HIV. In Canada, the HIV epidemic first impacted gay and bisexual men, while the epidemic in women lagged. Hence, these sites were chosen based on the epidemiology of having the largest proportion of both men and women aging with HIV. When the CHANGE HIV study was first initiated, there were 972 people living with HIV, including 102 women, aged 65 years and older, who were actively receiving care in these clinics. An additional 1076 people living with HIV (256 women) at these centers were aged 60–64 years, with many becoming eligible for recruitment since the study initiation. To be eligible, the participants need to be able to sufficiently communicate in English or French to provide informed consent and complete study procedures. Potentially qualifying individuals are identified according to age with consecutive patients approached during routine clinic visits by the study site coordinator for participation and to obtain written informed consent. The participants receive compensation to cover the cost of transportation or parking at a rate of CAD 50 per study visit. The study received ethical approval from the University Health Network Research Ethics Board at the University of Toronto and from the individual study sites. All the participants provided written informed consent prior to any study activities.

### 2.3. Data Collection

To comprehensively describe the nature and extent of the health of people aging with HIV in Canada, a series of measures are collected at 18-month intervals by self-reporting, questionnaires, objective measures, and biomarkers (Tables S1 and S2). The goal is to collect 2–3 sets of evaluations per participant to describe changes in health over time. Each set of evaluations is administered over 3 visits to reduce cognitive fatigue and allow research coordinators to build rapport with study participants by the time more sensitive topics are evaluated (e.g., sexual satisfaction). On average, each study visit requires between 15 and 75 min to complete. The data collection is standardized across sites, utilizing standardized operational procedures developed by the project manager, who trains the site staff in the administration of the tools. During the first 2 years of the study, the majority of assessments were coordinated with routine in-person clinical visits and completed with the research coordinator on site. However, since the start of the COVID-19 pandemic, where possible, assessments were virtually conducted with the participants completing questionnaires online, over the phone, or using video with a research coordinator. 

In the CHANGE HIV cohort, health is evaluated across 7 domains, including chronic disease, mental health, pain, social support, quality of life, cognitive function, and physical function using measurement tools outlined in Table S1 [6,7,8,9,10,11,12,13,14,15,16,17,18,19,20,21,22,23,24,25,26,27,28,29,30]. In addition to this, a healthy aging score is calculated using a The Rotterdam Healthy Aging Score [30]. The data on determinants of health, including behavioral, economic, social, health and social services, physical environment, and personal factors are collected through self-report and questionnaires (Table S2) [31,32,33,34,35,36,37,38,39,40,41,42,43]. HIV-related parameters are assessed using chart reviews and laboratory testing as part of routine clinical care, including an in-depth evaluation of antiretroviral therapy exposure history and adherence, illness duration, exposure category, opportunistic infections, and other complications. The data on concomitant prescription, over-the-counter, and herbal medication use are collected in addition to vaccination history and use of facial fillers. All components of medical history are corroborated through a review of the medical record, in addition to the personal report. The participants have the option of contributing to a sub-study by providing blood samples and nasal and rectal swabs to assess for correlation between the microbiome and markers of immune activation with healthy aging. 

### 2.4. Data Management

The CHANGE HIV data are collected using web-based electronic case report forms and entered directly into a REDCap™ database either by the participants or site personnel. The database is housed at the Applied Health Research Centre (AHRC) of Unity Health in Toronto, Ontario. The AHRC, along with the study project manager, manage the database access and provide training to site personnel on data entry and verification through computerized and manual queries to ensure data quality.

## 3. Results

### 3.1. Enrolment, Retention and Mortality Rate

As of November 2022, a total of 353 participants were enrolled in the CHANGE HIV study, with the majority of participants recruited from study sites located in Ontario (79%) and from tertiary clinical sites (72%). A total of 2.8% of the participants had withdrawn from active data collection, with the most common reasons for withdrawal being losing interest in participation, being lost to follow up, and change in location. An additional 2.5% of the participants had died since their baseline assessment.

### 3.2. Participant Demographics

Table 1 provides an overview of baseline sociodemographic characteristics of the first 353 participants enrolled in the CHANGE HIV study. There are 323 cis-gender men, 27 cis-gender women, and 3 trans-gender women enrolled in the cohort. The baseline data are presented according to gender, with cis- and trans- women combined into a single category. The median and interquartile range (IQR) age at the time of enrolment was 70 years (67, 73) with a range from 65 to 85 years old. Reflecting the HIV epidemic evolution in Canada, most participants are male (91%), white (77%), and identify as men who have sex with men (MSM) (77%). However, among women, only 53% identified as white, and the majority (60%) were born outside of Canada. 

Overall, the men in the cohort tended to have a higher level of education and gross annual household income than the women, with nearly a third of women living on an income of less than CAD 20,000 per year. A total of 6% of women and 39% of men reported being in a relationship, with higher rates of divorce or separation (27%), and being widowed (30%) reported by women. Nearly three quarters of women and half of men reported living alone.

The median (IQR) number of years since HIV diagnosis at the time of enrolment was 26 (19, 31) with a range from 1 to 41 years. The median [IQR] CD4 count nadir was 191 cells/mm^3^ (91, 324) with a range from 0 to 1500 cells/mm^3^. The CD4 nadir was lower among women, who reported a median CD4 count nadir of 160 cells/mm^3^, with none of the women having a nadir of ≥500 cells/mm^3^.

### 3.3. Participant Medical Background

Table 2 summarizes the most commonly reported comorbidities among the CHANGE HIV cohort participants as well as hepatitis B and C co-infection status and smoking and alcohol use history. Only 5% of men and 10% of women reported no comorbidities. History of cancer, coronary artery disease, chronic kidney disease, history of addiction, and prior stroke were more common among men. In contrast, arthritis, peripheral neuropathy, osteoporosis, depression/anxiety, and Human Papillomavirus (HPV)-related disease were more common among women. The median (IQR) body mass index (BMI) was 25.6 kg/m^2^ (23.4, 28.4) among men and 27.1 kg/m^2^ (24.0, 32.2) among women, with a third of women and 17% of men in the obesity range (BMI ≥ 30 kg/m^2^). About a third of men and a fifth of women had a positive hepatitis B core antibody and one in ten had positive hepatitis C antibody. Current and prior smoking history was more common among men and men had higher daily alcohol consumption than women. 

### 3.4. Participant Frailty, Cognitive Function, Loneliness, and Resilience

There was a high prevalence of pre-frailty and frailty among CHANGE HIV cohort participants, with only 19% of men and 36% of women meeting the threshold for non-frail or robust phenotype as per the Fried Frailty Phenotype [6]. Women had worse cognitive function scores than men (Table 3) on the Mini Mental Status Evaluation (MMSE) [23]. Men and women reported a similar degree of loneliness on the UCLA loneliness scale where loneliness is measured on a scale of 20–80 (higher scores corresponding to greater degree of loneliness), with a median [IQR] score of 27 (22, 39) among men and 28 (23, 44) among women [30]. This suggests a lower degree of loneliness than reported among older adults in the general population (median scores 30.5–33) [29,44] who were of similar age to those in the CHANGE HIV study but with a higher proportion of women. There were no gender differences in resilience scores using the Connor-Davidson Resilience Scale, and the scores were similar to those reported among older adults in the general population [39,45]. 

## 4. Discussion

Our preliminary results from the CHANGE HIV cohort demonstrate that older adults living with HIV in Canada are a heterogenous group, including long-term survivors who have aged with HIV and those who were infected at an older age. The participants come from a variety of social backgrounds, psychosocial pressures, and lived experiences, which may ultimately influence how they age. Several potential vulnerabilities among cohort participants have been observed, including a high prevalence of comorbidities and frailty/pre-frailty, financial insecurity (especially among women), and fragile social structures with a large proportion of individuals living alone. The majority of participants are single, divorced, or widowed, with only 6% of women married or in steady relationships. Despite this, the study participants reported a slightly lower overall degree of loneliness compared to older adults in the general population [29,44]. In a cross-sectional study of 356 persons living with HIV in San Francisco aged 50 years and older (median age 56), there was a similar prevalence of moderate and severe loneliness (22% and 12%, respectively) [46]. Loneliness was associated with functional impairment and lower quality of life, with those reporting loneliness being more likely to have depression and substance use. This suggests that mental health and psychosocial assessments and interventions may be necessary to improve overall health outcomes in older adults living with HIV.

Interpersonal factors may have significant downstream effects on the resources available to individuals aging with HIV. Social structures, including housing and marital status, must be considered as we strive to develop sustainable and equitable access to care and align our healthcare system with the needs of the aging HIV population. Long-term care facilities may be particularly challenged by the rising number of persons living with HIV entering care, given their lack of experience of working with this population and managing their complex social and health care needs, thus putting persons living with HIV at risk of stigma and discrimination.

In the CHANGE HIV cohort, the majority (64%) of individuals were deemed pre-frail according to the Fried Frailty Phenotype, with 15% categorized as frail. There have been several proposed ways of defining the clinical syndrome of frailty, causing comparisons between populations to be challenging [6,47,48]. Overall, frailty represents an individual’s vulnerability to various physical, cognitive, social, and emotional stressors as a result of the progressive loss of physiological reserves. Frailty is an important predictive measure shown to be associated with increased risk of falls, disability, hospitalization, and mortality [6]. The Fried Frailty Phenotype is one of the most commonly used measures, which focuses on physical frailty according to five criteria (unintentional weight loss, exhaustion, weakness, slow walking speed, and low physical activity), and has been shown to be associated with older age and multimorbidity. In the Cardiovascular Health Study of over 5300 individuals from the general population, age 65 and older (mean age 72), 7% were deemed to be frail, with the prevalence increasing from 3.2% in the 65–70 group to 23.1% in the 90+ group [6]. Nearly half were pre-frail (47%). The prior estimates of frailty among people living with HIV in the Dutch AGEhIV cohort also found a greater prevalence of frailty phenotype (10% compared to 3% of HIV-negative controls) [49]. The higher prevalence of frailty in our cohort is likely a reflection of older age among study participants (median age of 70 in our cohort, compared to the mean age of 52 in the Dutch study). The high rates of polypharmacy, especially the use of anticholinergic drugs, among older adults living with HIV seems to be one of the contributing factors to the high prevalence of frailty in this population [50]. Overall, these findings highlight the importance of understanding how pre-frailty evolves into frailty and what strategies can help delay or mitigate this progress among those aging with HIV.

Our study builds on the knowledge gained from other cohorts looking at the effects of aging with HIV, where older age was historically considered to be >45–50 years old. For example, the United States-based Veterans Aging Cohort Study (VACS) compares clinical outcomes between >40,000 veterans living with HIV and 1:2 age-, race-, and site-matched HIV negative controls, with a median age of 53 years old [51]. The generalizability of the findings to the aging HIV population in Canada is limited given the almost complete focus on male veterans. Another study from the United Sates, Research on Older Adults with HIV (ROAH), is a study of nearly 1000 persons living with HIV in New York City [52]. Although there is a better representation of women and transgender persons in the cohort (30%), the average age of participants is 55 with only 16% in the 60+ age group. Furthermore, the geographic constraint of the study causes it to be less generalizable to the Canadian population. The AGEhiV cohort in the Netherlands compares comorbidity among 500 people living with HIV (85% men) over the age 45 to HIV-negative controls, enrolled from sexually transmitted disease clinics [53]. With only 11% of participants in the 65+ age group, there are limitations to understanding multi-morbidity in older people living with HIV. Similarly, in the Pharmacokinetic and Clinical Observations in People Over Fifty (POPPY) study, the individuals living with HIV over and under the age 50 are enrolled from clinical sites in England and Ireland and compared to HIV-negative controls over the age 50 [54]. The median age of those in the older HIV-positive cohort is 56 (88% men). In contrast, our study focuses exclusively on older adults with HIV, age 65 and older, providing important insights into the factors that support healthy aging among those living with HIV beyond the evaluation of comorbidities. 

The main strength of the CHANGE HIV cohort is the multidimensional, comprehensive, and intersectional approach it adopts toward health and aging, informed by priorities identified though community engagement. The study is building a strong foundation for future research looking to understand what factors influence health outcomes in persons living with HIV, thus creating a platform for the development of interventional strategies to optimize aging with HIV. Unlike international cohorts established to study aging in HIV in those over ages from 45 to 50 [51,52,53,54], the CHANGE HIV cohort focuses on older adults, which is reflective of the demographic shift in the population as life expectancy among persons living with HIV approaches that of the general population [4]. As such, the CHANGE HIV cohort brings forward new knowledge and allows comparisons to be made to older adults in the general population. 

The weaknesses of the cohort include potential recruitment bias with underrepresentation of certain groups (e.g., indigenous peoples, new immigrants, those residing in long-term care facilities, those not engaged in care, or people living in rural or remote communities) since participants are enrolled from urban clinical sites and are required to complete the study procedures in either English or French. Although reflective of the aging HIV epidemic in Canada, there is an underrepresentation of women in the cohort. The preliminary results from the study already demonstrate important differences in health determinants and outcomes between genders and, therefore, as the cohort continues to enroll, there is an impetus to increase gender diversity. The cohort recruitment was impacted by the COVID-19 pandemic. Many financial and social impacts of the pandemic could confound the effects of aging with HIV. In future analyses, we will compare the results of many of the measures before and after the pandemic and a COVID-19 sub-study will more directly address some of these issues.

In many ways, the creation of the first Canadian geriatric HIV cohort of those aged 65 years and older is a celebration of the advances in HIV care, with many people living with HIV now living into advanced age. It is also an opportunity for us to understand what the aging process looks like among those living with HIV, including long-term survivors and those who were infected at an older age, and what strategies will empower persons living with HIV and their caregivers to optimize health, quality of life, and independence. The CHANGE HIV study team encourages collaborators to submit proposals for analysis projects. Any requests for data can be delivered to the CHANGE HIV steering committee.

## Figures and Tables

**Table 1:** Sociodemographic and baseline HIV characteristics of participants enrolled in the CHANGE HIV cohort, according to gender.

Participant Characteristics	Men	Women
	(*n* = 323)	(*n* = 30)
Age at enrolment (years)		
65–69	49%	57%
70–74	35%	33%
75–79	13%	7%
≥80	3%	3%
Race/ethnicity		
White	79%	53%
Black	9%	37%
Indigenous	0.3%	0%
Asian	3%	7%
Hispanic	3%	0%
Other	6%	3%
Canadian born	66%	40%
Highest level of education completed		
Elementary school	5%	23%
Secondary school diploma or equivalent	19%	27%
Apprenticeship or trades certificate	3%	3%
College or other non-university certificate	18%	20%
University certificate or diploma	9%	7%
Bachelor’s degree	24%	10%
Master’s degree	17%	7%
PhD	4%	3%
Retired	74%	73%
Gross Annual Household Income (CAD)		
Under CAD 20,000	17%	31%
CAD 20,000–49,999	35%	34%
CAD 50,000–99,999	28%	31%
CAD ≥100,000	20%	3%
Marital status		
Single	42%	37%
Divorced or separated	11%	27%
Widowed	8%	30%
Steady partner	7%	0%
Common law partner	16%	3%
Married	16%	3%
Living arrangement		
Alone	53%	73%
With spouse/partner	38%	10%
With roommate	7%	0%
With friends	1%	3%
With family	2%	10%
Other	1%	3%
HIV exposure category		
Same sex only	83%	10%
Heterosexual only	11%	73%
Injection drug use only	1%	3%
Sex and injection drug use	2%	0%
Blood products	3%	13%
Duration of HIV infection at enrolment (years)		
<10	5%	13%
10–19	19%	27%
20–29	38%	43%
≥30	37%	17%
CD4 nadir (cells/mm^3^)		
<100	26%	21%
100–199	24%	42%
200–299	21%	25%
300–399	9%	8%
400–499	9%	4%
≥500	11%	0%

**Table 2:** Medical comorbidities and risk factors among participants enrolled in the CHANGE HIV cohort, according to gender.

Comorbidities and Risk Factors	Men (*n* = 323)	Women (*n* = 30)
Dyslipidemia	52%	50%
Hypertension	42%	40%
Cancer	32%	13%
Diabetes	24%	23%
Arthritis	21%	30%
Coronary Artery Disease	17%	13%
Peripheral Neuropathy	16%	23%
Liver Disease	16%	17%
Osteoporosis	15%	20%
Depression and/or Anxiety	15%	20%
Chronic Obstructive Lung Disease and/or Asthma	14%	14%
Chronic Kidney Disease	11%	7%
Substance Use Disorder	8%	3%
Human Papillomavirus-related Disease	8%	13%
Stroke	5%	3%
Body Mass Index		
<18.5 (Underweight range)	2%	4%
18.5 to <25 (Healthy Weight Range)	41%	32%
25 to <30 (Overweight Range)	40%	32%
≥30 (Obesity Range)	17%	32%
Hepatitis B Core Antibody Positive	31%	19%
Hepatitis C		
Antibody positive	10%	11%
RNA positive	5%	11%
Tobacco Smoking History		
Current	13%	3%
Past	50%	33%
Never	37%	63%
Alcohol Use		
None	23%	23%
Few Times Per Week or Less	58%	70%
Daily	19%	7%

**Table 3:** Baseline frailty, cognitive function, loneliness, and resilience scores among participants enrolled in the CHANGE HIV cohort, according to gender.

Measure	Men	Women
Fried Frailty Phenotype	(*n* = 290)	(*n* = 28)
Non-frail	19%	36%
Pre-frail	65%	57%
Frail	16%	7%
MMSE	(*n* = 295)	(*n* = 27)
30–26 Normal	94%	74%
25–20 Mild	5%	26%
<20 Moderate-severe	1%	0%
UCLA loneliness scale	(*n* = 255)	(*n* = 27)
Low degree of loneliness	67%	67%
Moderate degree of loneliness	19%	15%
Moderately high degree of loneliness	7%	18%
High degree of loneliness	6%	0%
Connor-Davidson Resilience Scale	(*n* = 255)	(*n* = 27)
Median score (IQR)	32 (27, 36)	32 (27, 36)

MMSE = Mini-Mental State Examination; UCLA = University of California, Los Angeles; IQR = Interquartile range.

## Data Availability

Data is not publicly available due to privacy restrictions.

## Supplementary Materials

The following supporting information can be downloaded at: https://www.mdpi.com/article/10.3390/v15020517/s1, Table S1: Domains of health measured in the CHANGE HIV cohort; Table S2. Determinants of health evaluated in the CHANGE HIV cohort.
