# Supplementary material for: Correlates of Healthy Aging in Geriatric HIV (CHANGE HIV)—CTN 314
Source: Viruses. 2023 Feb 13;15(2):517. doi: 10.3390/v15020517 (PMC9968122; doi:10.3390/v15020517)
Supplement: Supplementary file 1 [file viruses-15-00517-s001.zip › viruses-2168543-supplementary.pdf]

Supplementary tables:

Table S1. Domains of health measured in the CHANGE HIV cohort.

**Domain of health**	**Measure**	**Measurement tools**
Physical function	Frailty<br><br>Disability<br>Independence<br><br>Falls<br>Urinary incontinence<br>Insomnia<br>Vision<br>Hearing<br>Fatigue	Fried frailty phenotype [6]<br>Frailty Index [7]<br>HIV Disability Questionnaire [8]<br>Basic Activities of Daily Living [9]<br>Instrumental Activities of Daily Living [10]<br>ACTG 5322 falls questionnaire [11]<br>Bladder diary [12]<br>Pittsburg sleep quality index [13]<br>Snellen vision chart [14]<br>Hearing handicap inventory for the elderly [15]<br>Functional Assessment of Chronic Illness Treatment fatigue scale [16]
Mental health	Anxiety<br>Depression	Hamilton Anxiety Rating Scale [17]<br>Beck Depression Scale [18]<br>Geriatric Depression Scale [19]<br>Center for Epidemiologic Studies Depression Score [20, 21]
Cognitive function	Learning, judgement, memory	Mini Mental Status Evaluation [22]<br>Montreal Cognitive Assessment (MoCA) [23]
Quality of life	Well being<br>Satisfaction	Life satisfaction questionnaire [24]<br>EQ5D [25]<br>MOS HIV [26]
Social supports	Social support networks<br>Loneliness	Duke social support index [27]<br>Medical outcome study social support survey [28]<br>UCLA Loneliness Scale [29]
Pain	Pain severity with activity and at rest	Rotterdam Healthy Aging Score pain sub-scale [30]
Chronic disease	Comorbidity	Chart review and self-report

UCLA= University of California, Los Angeles.

Table S2. Determinants of health evaluated in the CHANGE HIV cohort.

**Determinants of health**	**Measure**	**Measurement tool**
Behavioural	Coffee/Tea consumption<br>Food security<br>Sexual activity<br><br>Sexual satisfaction<br><br>Exercise<br>Smoking, Alcohol and other Substance use	Self-report- adapted from Liver cohort [31]<br><br>Food security survey adapted from OCS [32]<br>Questionnaire from OCS cohort for men [32]<br>Questionnaire from CHIWOS for women [33]<br>New sexual satisfaction questionnaire [34]<br>PROMIS sexual function and satisfaction tool [35]<br>Questionnaire adapted from OCS [32]<br>Questionnaire from CTN 222 [36]
Economic	Income and source<br>Work- current and past<br>Social security, pension	Scale of Economic Self-Sufficiency [37]<br>Self-report adapted from OCS [32]<br>Self-report adapted from OCS [32]
Social	Community engagement<br>Resilience<br>Violence and abuse<br>Stigma<br>Isolation, social network	Meaningful Activity Participation Assessment [38]<br>Resilience Scale [39]<br>Modified WHO IPV Scale (CHIWOS) [33]<br>HIV stigma scale [40]<br>NIH Toolbox Adult Social Relationship Scales [41]
Health and social services	Primary/specialty care<br>Mental health support<br>Addictions counselling<br>Physiotherapy and Rehabilitation<br>AIDS service organizations<br>Long term care<br>Hospitalization	Self-report adapted from OCS and CTN222 [32, 36]
Physical environment	Urban or rural<br>Transportation<br>Housing<br>Household members	Self-report
Personal	Age<br>Biologic sex<br>Education level<br>Literacy<br>Marital status<br>Children<br>Ethnicity	Self-report

	Year of immigration<br>Gender role<br>Gender identity	 Bem Sex role inventory short form [42]<br>Sexual Identity Scale [43]

OCS= Ontario HIV Treatment Network Cohort Study; CHIWOS= Canadian HIV Women's Sexual and Reproductive Health Cohort Study; CTN= Canadian Institutes of Health Research Canadian HIV Trials Network; NIH= National Institutes of Health.
